# PMRVT: Parallel Attention Multilayer Perceptron Recurrent Vision Transformer for Object Detection with Event Cameras

**DOI:** 10.3390/s25216580

**Published:** 2025-10-25

**Authors:** Zishi Song, Jianming Wang, Yongxin Su, Yukuan Sun, Xiaojie Duan

**Affiliations:** 1 School of Electronics and Information, Tiangong University, Tianjin 300387, China; 2331081031@tiangong.edu.cn; 2 Tianjin Key Laboratory of Autonomous Intelligence Technology and Systems, Tiangong University, Tianjin 300387, China; wangjianming@tiangong.edu.cn; 3 School of Computer Science and Technology, Tiangong University, Tianjin 300387, China; tgu_syx@163.com; 4 Center for Engineering Internship and Training, Tiangong University, Tianjin 300387, China; sunyukuan@tiangong.edu.cn; 5 School of Control Science and Engineering, Tiangong University, Tianjin 300387, China

**Keywords:** vision transformer, event camera, object detection, attention mechanism

## Abstract

Object detection in high-speed and dynamic environments remains a core challenge in computer vision. Conventional frame-based cameras often suffer from motion blur and high latency, while event cameras capture brightness changes asynchronously with microsecond resolution, high dynamic range, and ultra-low latency, offering a promising alternative. Despite these advantages, existing event-based detection methods still suffer from high computational cost, limited temporal modeling, and unsatisfactory real-time performance. We present PMRVT (Parallel Attention Multilayer Perceptron Recurrent Vision Transformer), a unified framework that systematically balances early-stage efficiency, enriched spatial expressiveness, and long-horizon temporal consistency. This balance is achieved through a hybrid hierarchical backbone, a Parallel Attention Feature Fusion (PAFF) mechanism with coordinated dual-path design, and a temporal integration strategy, jointly ensuring strong accuracy and real-time performance. Extensive experiments on Gen1 and 1 Mpx datasets show that PMRVT achieves 48.7% and 48.6% mAP with inference latencies of 7.72 ms and 19.94 ms, respectively. Compared with state-of-the-art methods, PMRVT improves accuracy by 1.5 percentage points (pp) and reduces latency by 8%, striking a favorable balance between accuracy and speed and offering a reliable solution for real-time event-based vision applications.

## 1. Introduction

Object detection in high-speed and dynamic scenarios remains a critical challenge in computer vision, with broad applications in autonomous driving, robotics, and surveillance. Conventional frame-based cameras are limited by fixed frame rates and narrow dynamic ranges, often resulting in motion blur and missed detections. While recent event-based methods partially address these issues, they still face substantial challenges in computational efficiency and temporal modeling [1,2,3].

Event cameras, inspired by biological retinas, capture brightness changes asynchronously at the pixel level, providing high dynamic range, microsecond-level temporal resolution, and sub-millisecond latency [4,5]. These properties allow reliable object detection in rapidly changing scenes by maintaining spatial and temporal fidelity during fast motion. Compared with traditional cameras, event cameras reduce the computational burden associated with redundant frame processing and enhance responsiveness in real-time applications [6].

Despite these advantages, current event-based object detection approaches such as CNN-based models [7,8], Graph Neural Networks (GNNs) [9], Spiking Neural Networks (SNNs) [10], and Transformer-based architectures [11] still encounter critical limitations. CNNs are effective for spatial feature extraction but often lack explicit temporal modeling and are computationally intensive. GNNs can capture spatiotemporal correlations, as in AEGNN [12] and MGLNN [13], but suffer from scalability issues. SNNs like CREST [14] are biologically plausible and energy-efficient, yet difficult to train at depth, hindering their scalability. Transformer-based methods such as RVT [11] enhance long-range temporal dependency modeling, but the quadratic complexity of their self-attention mechanisms results in high latency and computational cost, restricting real-time deployment.

To address these challenges, we propose the Parallel Attention Multilayer Perceptron Recurrent Vision Transformer (PMRVT), a systematic framework for event-based object detection that jointly optimizes computational efficiency, spatial representation capacity, and temporal consistency. Its core design is based on the following three complementary innovations:

- Hybrid Hierarchical Feature Extraction Backbone: PMRVT adopts a progressive backbone that balances efficiency with expressiveness. Shallow stages employ streamlined feedforward operations to efficiently encode sparse event inputs at low computational cost, while deeper layers introduce attention-based modules to capture long-range dependencies and semantic abstraction. This design preserves tractability in early stages while enabling rich global context modeling at deeper levels.
- Parallel Attention Feature Fusion (PAFF): To enhance spatial expressiveness, PMRVT introduces a Parallel Attention Feature Fusion mechanism realized through a coordinated dual-path attention design. This design jointly models local fine-grained dependencies and global contextual abstraction, aligning and fusing multi-scale event patterns while structurally reconciling efficiency with global modeling capacity. This enables robust detection across diverse object scales and event densities.
- Temporal Integration Module for Context-Aware Consistency: PMRVT incorporates a recurrent integration module augmented with enlarged receptive fields to capture long-horizon temporal dependencies in asynchronous event streams. By aggregating sparse signals over extended time spans, it adaptively models object motion trajectories and dynamic scene evolution, unifying temporal consistency with resilience to event sparsity for real-time event-based perception.

The main objective of this manuscript is to design and rigorously evaluate a novel architecture for efficient and accurate object detection from event streams, with explicit goals to improve detection accuracy, reduce computational complexity (FLOPs/parameters), and lower inference latency for real-time deployment. We conduct a comprehensive experimental study on two benchmark event-camera datasets, Gen1 and 1 Mpx, including ablation experiments, statistical stability analysis, and robustness tests under noise and event loss. The results show that PMRVT achieves 48.7% and 48.6% mAP on Gen1 and 1 Mpx, with inference latencies of 7.72 ms and 19.94 ms, respectively. Compared with state-of-the-art methods, PMRVT improves detection accuracy by 1.5 percentage points (pp) while reducing inference latency by 8%. These findings demonstrate that PMRVT consistently attains higher mAP with lower inference time than strong baselines, validating its suitability for real-time event-based perception and highlighting its practical value for time-critical applications such as autonomous driving and robotic vision.

## 2. Related Work

### 2.1. Object Detection Based on Event Cameras

**Figure 1:**
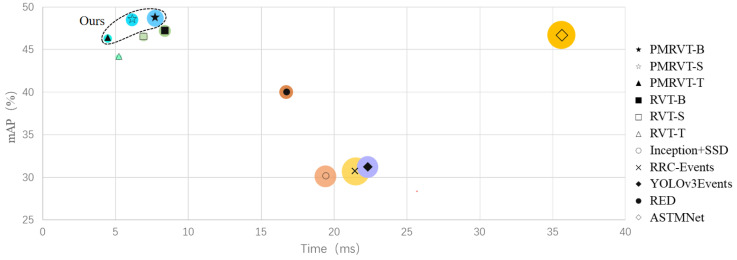
Accuracy–latency trade-off across representative event-based detectors on Gen1 and 1 Mpx. Each point denotes a method; the horizontal axis indicates per-frame inference time (ms), and marker size encodes parameter count (M). PMRVT achieves a favorable balance by improving mAP while reducing latency relative to strong baselines.

Event cameras, inspired by biological retinas, asynchronously detect changes in brightness and produce sparse streams of events with high temporal resolution, high dynamic range, and low latency. These properties make them well suited for object detection in dynamic and resource-constrained environments. However, their sparse and asynchronous output poses challenges for traditional frame-based deep learning models. As summarized in Figure 1, our method attains a favorable accuracy–latency trade-off compared with representative event-based detectors.

Early methods such as RED [7] and ASTMNet [8] convert events into dense representations like 2D histograms or voxel grids to enable compatibility with CNNs. While this facilitates feature extraction, it often sacrifices temporal resolution and introduces latency.

To better model temporal relationships, graph-based approaches such as AEGNN [12] and MGLNN [13] have been proposed. These methods capture event correlations at both local and global levels through spatiotemporal graph construction. Although effective, such graph models tend to be computationally intensive and are difficult to deploy in real-time systems.

Spiking Neural Networks (SNNs) provide another biologically inspired direction. CREST [14] demonstrated that spike-based temporal encoding can improve performance in event-based scenarios. Cordone et al. [15] further explored SNNs for object detection on GEN1 automotive data, achieving notable energy efficiency. Nonetheless, SNNs remain difficult to train and optimize, especially for deep architectures, and often underperform in terms of accuracy.

CNN-based models such as DenseSPH-YOLOv5 [16] utilize conventional backbones to achieve high detection accuracy. However, they typically lack explicit temporal modeling and are computationally demanding.

Hybrid architectures that combine CNNs and RNNs, such as ReYOLOv8s [17], incorporate recurrent layers to capture motion dynamics, achieving improvements in latency and temporal coherence. Meanwhile, Transformer-based methods have shown promise in modeling long-range dependencies. For example, EvRT-DETR [18] adopts a DETR-style Transformer augmented with recurrence, while FlexEvent [19] introduces ERGO-12 representations and frequency-adaptive learning to address diverse motion frequencies.

Peng et al. proposed several adaptive transformer variants tailored for event data. SAST [20] introduces sparse attention modules that adaptively focus on scene-relevant regions, reducing computational cost. However, this model depends heavily on accurate scene priors and remains relatively complex. Similarly, Cordone et al. [15] showed that spiking networks may offer efficiency advantages, but performance remains below transformer baselines.

In summary, existing methods either suffer from high computational costs or fail to fully exploit the unique spatiotemporal characteristics of event data. PMRVT systematically addresses these limitations through a unified framework that integrates three complementary principles: a Hybrid Hierarchical Feature Extraction Backbone for early efficiency, a Parallel Attention Feature Fusion (PAFF) mechanism with a coordinated dual-path attention design for enriched spatial expressiveness, and a Temporal Integration Module for context-aware long-horizon consistency. This design achieves strong accuracy and real-time performance on both Gen1 and 1 Mpx datasets, demonstrating a favorable trade-off between efficiency and precision.

### 2.2. Efficient Attention Strategies in Vision Transformers

Transformers are widely used in object detection for their ability to model long-range dependencies through self-attention. However, the standard multi-head self-attention (MSA) mechanism has quadratic computational complexity with respect to the input sequence length, making it impractical for high-resolution inputs in real-time applications.

To mitigate this, various attention strategies have been proposed. Hierarchical Vision Transformers (HVTs) [21,22] adopt pyramid-like architectures to reduce attention computation in early layers. Pan et al. [23] show that increasing the number of attention heads enhances MSA’s capacity to capture both local and global features. They also propose replacing early-stage attention with MLPs to reduce complexity, reintroducing self-attention in later stages where resolution is lower. Studies such as [24] further suggest that shallow ViT layers focus on local features while deeper layers handle semantic context.

Inspired by these insights, PMRVT adopts a hybrid strategy—employing MLPs for early-stage spatial encoding and using attention blocks only in deeper layers. This design significantly reduces computational burden while maintaining accurate spatiotemporal representations, striking a balance between efficiency and expressiveness.

## 3. Materials and Methods

### 3.1. Event Data Processing

Event cameras asynchronously emit a stream of sparse data, where each event indicates a significant brightness change at a pixel. To enable compatibility with standard convolutional networks, the raw event stream is converted into a dense, structured tensor representation.

Given a time window $\left[t_{a},t_{b}\right]$, events are first temporally normalized, as follows:

(1) $$\tau_{i}=T\cdot \frac{t_{i}-t_{a}}{t_{b}-t_{a}},$$

where $\tau_{i}\in \left[0,T\right]$ denotes the discretized time step and *T* is the total number of temporal bins.

The events are then aggregated into a tensor of shape [2T,H,W], where the first dimension encodes both polarity and time. For each pixel location, two channels per time step count the number of positive and negative events. This voxel grid preserves the spatiotemporal structure of the event data and enables efficient processing using 2D convolutions.

### 3.2. Hybrid Hierarchical Feature Extraction Backbone

**Figure 2:**
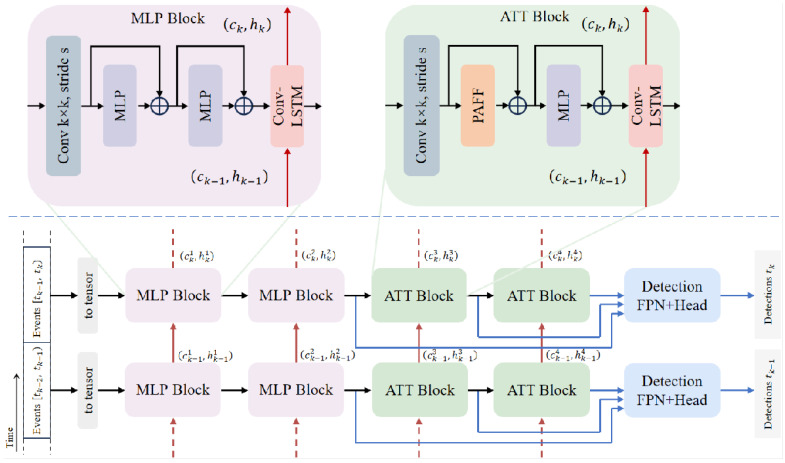
**PMRVT overview.** Shallow stages use lightweight MLP operations for efficient local encoding, deeper stages adopt the PAFF module to fuse block-wise and grid-wise self-attention in parallel, and a ConvLSTM temporal module aggregates spatiotemporal cues across event sequences.

To address the challenges of asynchronous and sparse event streams, PMRVT adopts a Hybrid Hierarchical Feature Extraction Backbone, as illustrated in Figure 2. Instead of applying uniform processing across all layers, this backbone is organized into a progressive hierarchy that balances shallow efficiency with deep expressiveness.

In the shallow stages, lightweight feedforward projections are employed in place of costly attention mechanisms. These localized operations efficiently encode fine-grained event patterns with minimal FLOPs and parameter count, ensuring tractable early-stage processing even under high event rates. As the hierarchy deepens, attention-based modules are introduced to enrich spatial representation, capturing long-range dependencies and semantic abstraction. This principled progression yields a balanced design: early layers emphasize efficiency and locality, while deeper layers deliver expressive global modeling.

### 3.3. ATT-Block: Parallel Attention Feature Fusion

**Figure 3:**
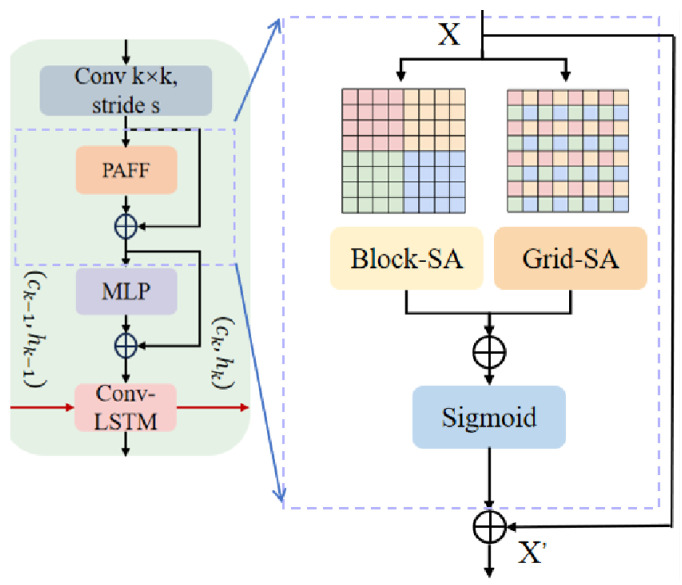
Parallel Attention Feature Fusion (PAFF). The input feature is processed by two parallel streams: block-wise self-attention for local and fine-grained dependencies.

The ATT-Block is the concrete module that realizes our proposed Parallel Attention Feature Fusion (PAFF) mechanism. A schematic of the module is shown in Figure 3. It enhances spatial representation by jointly capturing local fine-grained dependencies and global contextual abstraction. Unlike sequential attention designs that first process local and then global cues, the ATT-Block adopts a coordinated dual-path attention design in a structurally parallel layout, thereby reconciling efficiency with expressive capacity.

Formally, the fusion of local and global pathways is expressed as

(2) $$X^{\prime }=X+\sigma \left(B(X)+G(X)\right),$$

where *X* is the input feature map, σ denotes the Sigmoid activation function, B(X) is the block-wise self-attention output, and G(X) is the grid-wise self-attention output.

The block-wise pathway partitions the feature map into non-overlapping regions of size P×P, computing fine-grained dependencies, as follows:

(3) $$B\left(X\right)=\mathrm{Concat}\left[\mathrm{Softmax}\left(\frac{Q_{b}K_{b}^{T}}{\sqrt{d}}\right)V_{b}\right],$$

where $Q_{b},K_{b},V_{b}$ are queries, keys, and values projected from each block. This pathway emphasizes sensitivity to localized structural variations.

In parallel, the grid-wise pathway sparsely samples features with stride *G*, capturing long-range dependencies with reduced redundancy, as follows:

(4) $$G\left(X\right)=\mathrm{Interp}\left[\mathrm{Softmax}\left(\frac{Q_{g}K_{g}^{T}}{\sqrt{d}}\right)V_{g}\right],$$

where Interp restores global responses to full resolution, preserving fidelity while maintaining efficiency.

By coordinating these two streams, the ATT-Block operationalizes the PAFF mechanism, mitigating over-concentration on dominant activations and producing robust multi-scale spatial representations. Within PMRVT, it serves as the spatial refinement stage of the Hybrid Hierarchical Feature Extraction Backbone, complementing the shallow efficiency-preserving encoder and preparing features for temporal integration.

### 3.4. Temporal Integration Module for Context-Aware Consistency

Asynchronous event streams are inherently irregular and sparse, making it challenging to maintain temporal coherence across varying densities. To ensure robust modeling under these conditions, PMRVT incorporates a Temporal Integration Module for context-aware temporal consistency. This module extends recurrent modeling with convolutional operations, preserving spatial locality while explicitly aggregating temporal dependencies.

The hidden state update follows a gated convolutional formulation, as follows:

(5) $$C_{t}=f_{t}⊙C_{t-1}+i_{t}⊙\tanh\left(W_{xc}*x_{t}+W_{hc}*h_{t-1}\right),$$

(6) $$h_{t}=o_{t}⊙\tanh\left(C_{t}\right),$$

where ∗ denotes convolution, enabling information flow across both spatial and temporal dimensions.

By enlarging convolutional receptive fields, the module aggregates sparse signals across extended horizons, allowing robust modeling of object trajectories and dynamic scene evolution under varying event densities. Positioned after spatial fusion, it provides the temporal backbone that stabilizes long-horizon consistency in PMRVT.

### 3.5. Detection Head and Loss Function

**Figure 4:**
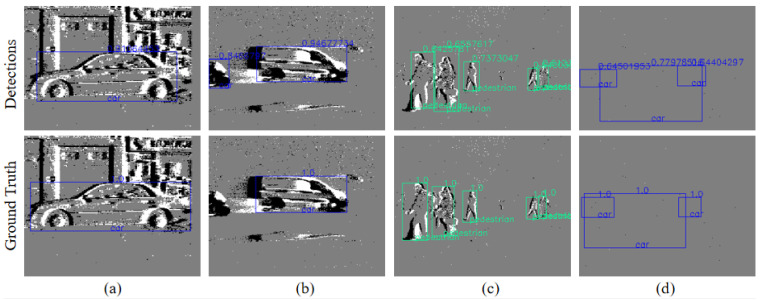
**Predictions on the Gen 1 dataset.** All examples are picked to illustrate the detection results of different-sized targets in the Gen1 dataset. (**a**–**c**) illustrate detection results for large, medium, and small targets, respectively, in the Gen1 dataset; (**d**) shows the situation where the model can successfully identify targets even when the number of events is insufficient for detection.

The final detection results are obtained using a YOLO detection head. Qualitative detection results on Gen1 are presented in Figure 4. The model employs the standard YOLO loss function, combining three components: bounding box regression loss ($L_{\mathrm{bbox}}$), objectness loss ($L_{\mathrm{obj}}$), and classification loss ($L_{\mathrm{cls}}$), as follows:

$$L_{\mathrm{total}}=\lambda_{1}L_{\mathrm{bbox}}+\lambda_{2}L_{\mathrm{obj}}+\lambda_{3}L_{\mathrm{cls}}$$

where $\lambda_{1},\lambda_{2},\lambda_{3}$ are empirically determined coefficients optimized during the training process. These losses guide the model to refine the bounding boxes, enhance object classification, and improve objectness prediction, ensuring accurate and efficient detection performance across dynamic environments.

### 3.6. Complexity Analysis

To complement the experimental evaluation, an asymptotic analysis of the computational complexity of PMRVT is conducted and compared with that of the baseline RVT. The complexity of key modules is summarized in Table 1. Let N=H×W denote the number of spatial tokens, *d* the feature dimension, *P* the block size, and *G* the grid stride.

In RVT, shallow layers employ global self-attention with complexity $O(N^{2}d)$, which incurs prohibitive cost when *N* is large. PMRVT addresses this limitation by replacing early attention with lightweight MLP-based projections of complexity $O(Nd^{2})$, which scale linearly with *N*, thereby avoiding the quadratic bottleneck in the initial stages.

For spatial modeling, PMRVT introduces the Parallel Attention Feature Fusion (PAFF) mechanism that combines block-wise and grid-wise self-attention in parallel. The block-wise branch partitions the feature map into non-overlapping P×P regions, reducing the cost to $O(NP^{2}+\frac{N}{P^{2}}d^{2})$. The grid-wise branch samples features with stride *G*, reducing the sequence length to $N/G^{2}$ and yielding complexity $O(\frac{N}{G^{2}}d^{2})$. Since the two branches operate in parallel, the total cost is additive while remaining near-linear in *N*, which is considerably lower than the quadratic $O(N^{2}d)$ cost of global attention in RVT.

This reduction from quadratic to near-linear complexity provides a theoretical explanation for the empirical results in Section 4, where PMRVT achieves lower latency and simultaneously improves accuracy compared with RVT.

## 4. Experiments

In this section, comprehensive experiments are conducted to assess the performance of the proposed PMRVT model against several baseline models. The primary objective is to evaluate the effectiveness of the PMRVT model in terms of accuracy, computational complexity, and inference time. This section is structured as follows: First, the experimental setup and implementation details are provided, followed by a description of the datasets used. Then, ablation studies are presented to analyze the contribution of different components of the model, such as the attention mechanism and LSTM configurations. Finally, benchmark comparisons with state-of-the-art models are provided to highlight the advantages of the proposed model in comparison with existing methods.

### 4.1. Setup

#### 4.1.1. Implementation Details

In the conducted experiments, all model layers were randomly initialized, except for the LayerScale, which was set to $1\times 10^{-5}$. The model was trained using the ADAM optimizer [25] with a OneCycle learning rate schedule [26], which linearly decays from the maximum rate.

The hardware environment included an Intel Core i7-10700K CPU (Intel Corporation, Santa Clara, CA, USA), 64 GB RAM, and NVIDIA GeForce RTX 3090 (24 GB VRAM) and RTX 3090 Ti (24 GB VRAM) GPUs (NVIDIA Corporation, Santa Clara, CA, USA). For the Gen1 dataset, training was performed on a single RTX 3090 GPU with batch size 8, sequence length 21, and learning rate $2\times 10^{-4}$, taking approximately 2 days. For the 1 Mpx dataset, training was conducted on two RTX 3090 Ti GPUs with batch size 24, sequence length 5, and learning rate $3.5\times 10^{-4}$, lasting about 5 days. Model evaluation was carried out on a single RTX 3090 GPU.

The software environment consisted of Ubuntu 20.04 LTS (Canonical Ltd., London, UK), CUDA 11.3, cuDNN 8.2, and PyTorch 1.10.

#### 4.1.2. Dataset

The experiments were conducted using two distinct event camera datasets: Gen1 and 1 Mpx. The Gen1 dataset comprises 750 GB of event stream data recorded across various environments over 39.32 h, containing 228,123 car labels and 27,658 pedestrian labels. The dataset is divided into 2359 samples: 1460 for training, 470 for testing, and 429 for validation. The 1 Mpx dataset, recorded across diverse locations, contains 14.65 h of data and is composed of approximately 25 million frames. Bounding boxes for the RGB video stream were generated using a commercial car detector, which identified pedestrians, bicycles, and cars for precise object recognition. The 1 Mpx dataset, like the Gen1 dataset, is used for evaluating the model’s generalization across different environments.

#### 4.1.3. Model Variants

For the experiments, three variants of the PMRVT model were trained: Base (B), Small (S), and Tiny (T). These variants differ in the number of channels and kernel sizes, providing a comprehensive comparison of the model’s performance under different configurations. The Base variant (PMRVT-B) uses the largest model configuration with the highest number of channels in each block, while the Small (PMRVT-S) and Tiny (PMRVT-T) variants reduce the number of channels to accommodate different trade-offs between accuracy and computational cost. The specific configurations for each variant, including kernel sizes, strides, and channel numbers for each block, are listed in Table 2. This allows us to assess how different model sizes and architectures impact the overall performance of the PMRVT model.

### 4.2. Benchmark Comparisons

This section presents a comparison of the proposed PMRVT model against representative state-of-the-art detectors for event-based object detection, including AsyNet [27], AEGNN [12], MatrixLSTM [28], RED [7], RT-DETR-B [18], S5-ViT-B [8], GET [29], AEC [30], SAM [30], Inception+SSD [31], RRC-Events [7], YOLOv3Events [32], ASTMNet [33], and the RVT baseline [11]. The quantitative results on the Gen1 [34] and 1 Mpx [35] datasets are summarized in Table 3.

The results show that PMRVT (3 × 3) achieves 48.7% mAP on the Gen1 dataset, surpassing RVT [11] (47.2%) and S5-ViT-B [8] (47.2%) by 1.5 percentage points (pp) and 1.2 pp, respectively, while also outperforming CNN/RNN-based approaches such as AsyNet [27], RED [7], and ASTMNet [33]. On the 1 Mpx dataset, PMRVT (3 × 3) reaches 48.6% mAP, which is 1.2 pp higher than RVT (47.4%). These results demonstrate that PMRVT outperforms existing methods in feature extraction capability, especially after incorporating parallel attention mechanisms and ConvLSTM.

In terms of computational complexity, PMRVT (1 × 1) requires 4.40 GMac, compared with RVT’s 5.1 GMac, reflecting higher computational efficiency. PMRVT (1 × 1) also uses 14.3 M parameters versus RVT’s 18.5 M, indicating reduced model size while maintaining high performance.

Regarding inference time, PMRVT (3 × 3) achieves 7.72 ms on Gen1, compared with RVT’s 8.4 ms, and 19.94 ms on 1 Mpx, compared with RVT’s 22.61 ms. Despite PMRVT (3 × 3) having more parameters and higher FLOPs, it runs faster. This efficiency stems from the Parallel Attention Feature Fusion (PAFF) module—which computes local and global attention in parallel—and the hybrid design that uses lightweight MLPs in shallow stages while reserving attention modules for deeper, lower-resolution stages.

Overall, across both datasets, PMRVT delivers a favorable accuracy–efficiency trade-off, achieving higher mAP than RVT [11] and other CNN/RNN or Transformer baselines [7,8,12,18,27,28,29,30,31,32,33], while also reducing latency. Detailed results can be found in Table 3.

### 4.3. Ablation Study

This section presents a systematic ablation study to evaluate the contribution of each core component in the proposed PMRVT architecture. Specifically, we investigate three key aspects: the hierarchical hybrid progressive design, the efficiency gains from the parallel attention mechanism, and the spatiotemporal modeling capabilities of the ConvLSTM. All experiments are conducted on the Gen1 [34] validation set, using the best-performing checkpoint after 400,000 training iterations.

#### 4.3.1. Impact of Hierarchical Hybrid Progressive Architecture

PMRVT adopts a hierarchical hybrid progressive architecture that uses lightweight MLP blocks in the shallow stages and attention mechanisms in the deeper stages. This design optimizes the trade-off between computational cost and detection performance.

We tested four configurations by replacing the first two RVT-style blocks with MLP blocks. The results in Table 4 show that substituting RVT blocks with MLP blocks reduces parameters from 18.52 M to 14.26 M and FLOPs from 5.07 GMac to 4.40 GMac, while maintaining or slightly improving mAP. The best configuration (MLP in the first two stages and ATT in the last two) achieves the highest mAP of 47.7%, representing a 0.5% improvement over the RVT baseline [11].

By using MLPs in early stages, we simplify the structure, reduce redundancy, and better align with the SIMD execution model of modern GPUs. Introducing the parallel attention mechanism in later stages (block-wise and grid-wise self-attention in parallel) further enhances efficiency. Overall, these results confirm that early MLP + late attention reduces redundancy and computational cost while preserving accuracy.

#### 4.3.2. Efficiency Improvement of Parallel Attention Mechanism

PMRVT’s Parallel Attention Feature Fusion (PAFF) enhances efficiency by handling local and global dependencies through two parallel paths: block-wise self-attention (Block-SA) and grid-wise self-attention (Grid-SA). Compared with the traditional sequential attention, this parallel structure reduces resource contention.

As shown in Table 4, enabling PAFF reduces parameters from 18.19 M to 14.26 M and FLOPs from 4.74 GMac to 4.40 GMac, while improving inference time from 6.84 ms to 6.40 ms. These gains stem from executing Block-SA and Grid-SA concurrently, thereby avoiding sequential bottlenecks found in RVT-style attention stacks [11]. This validates that PAFF delivers measurable efficiency and latency improvements by parallelizing local–global attention.

#### 4.3.3. Efficiency and Effectiveness of ConvLSTM Integration

To evaluate the effect of convolutional kernel size in the temporal module, we compare three configurations under the PMRVT framework: the RVT model with 1 × 1 LSTM [11], PMRVT with 1 × 1 ConvLSTM, and PMRVT with 3 × 3 ConvLSTM (Table 5).

Relative to RVT [11], PMRVT (1 × 1) reduces parameters from 18.5 M to 14.3 M and FLOPs from 5.1 GMac to 4.40 GMac, while reducing latency from 8.4 ms to 6.38 ms (a 24% improvement) and achieving 47.7% mAP, which is 0.5 percentage points (pp) higher than RVT [11]. Using 3 × 3 kernels increases parameters and FLOPs to 36.6 M and 9.77 GMac and latency to 7.72 ms, while attaining the highest accuracy of 48.7% mAP, representing a 1.5 pp improvement over RVT [11]. Despite the increased computational cost, PMRVT (3 × 3) remains faster than RVT (7.72 ms vs. 8.4 ms), demonstrating that the lightweight and parallel design accommodates stronger temporal modeling without compromising responsiveness. These results confirm that ConvLSTM substantively enhances spatiotemporal modeling, with 3 × 3 kernels offering the best balance between accuracy and efficiency.

#### 4.3.4. Impact of ConvLSTM Across Hierarchical Stages

We further evaluate the impact of ConvLSTM placement across the hierarchical stages of PMRVT (Table 6). Progressively moving ConvLSTM from the deepest stage (S4) toward earlier stages yields consistent improvements in detection accuracy. Applying ConvLSTM only at S4 achieves 37.5% mAP, extending it to S3–S4 increases performance to 41.5%, further integration into S2–S4 reaches 47.0%, and distributing ConvLSTM across all stages (S1–S4) achieves the highest accuracy of 48.7% mAP. These findings indicate that comprehensive spatiotemporal modeling across multiple resolutions is essential for event-based detection. Moreover, the observed gains corroborate the improvements over RVT [11] and validate that full-stage ConvLSTM integration is necessary for attaining optimal accuracy.

### 4.4. Robustness and Stability Analysis

#### 4.4.1. Stability Across Random Seeds

To ensure that the reported improvements are not caused by stochastic variation, we repeated each experiment with five different random seeds on both Gen1 and 1 Mpx. For each independent run, we report the mean ± standard deviation (SD) and estimate 95% confidence intervals (CIs) using stratified bootstrap with 1000 resamples. Furthermore, we conducted paired bootstrap tests under identical resampling indices to evaluate whether the improvements of PMRVT over RVT are statistically significant. Compared with single-run reporting, this protocol provides a more rigorous assessment of stability against random initialization and training dynamics.

Table 7 reports the results across multiple runs. On Gen1, PMRVT-3 × 3 achieves 38.7 ± 0.5 mAP, compared with 37.2 ± 0.4 for RVT, and the difference is statistically significant (p=0.008). On 1 Mpx, PMRVT-3 × 3 attains 43.0 ± 0.5 mAP versus 41.8 ± 0.6 for RVT, corresponding to a statistically significant improvement of +1.2 mAP (p=0.017). The non-overlapping confidence intervals further indicate that the observed improvements are unlikely to be attributable to random variation. Overall, these results demonstrate that the proposed architecture consistently outperforms the baseline while maintaining robustness under stochastic training variability.

#### 4.4.2. Robustness to Noise and Event Loss

To further examine robustness under sensor imperfections, we tested PMRVT with two stress conditions: (i) additive sensor noise, simulated by randomly perturbing 10% of events with Gaussian noise, and (ii) event loss, simulated by randomly dropping 20% of input events. Table 8 reports the results on the Gen1 dataset.

The results indicate that PMRVT consistently outperforms RVT under both noisy and incomplete event streams, demonstrating that its spatiotemporal modeling enhances resilience to real-world sensor imperfections.

## 5. Conclusions and Discussion

This paper has presented the Parallel Attention Multilayer Perceptron Recurrent Vision Transformer (PMRVT), a model developed for real-time event-based object detection in dynamic environments. By integrating lightweight MLP blocks in the shallow stages, parallel attention mechanisms in the deeper stages, and ConvLSTM modules for temporal modeling, PMRVT achieves an effective trade-off between computational efficiency and representational capacity. Extensive experiments on the Gen1 and 1 Mpx benchmarks demonstrate that PMRVT consistently surpasses strong baselines in terms of both accuracy and inference latency.

Nevertheless, several limitations remain. The evaluation has been confined to the Gen1 and 1 Mpx datasets, which, although widely used, predominantly represent urban and automotive scenarios and therefore do not comprehensively reflect the diversity of potential application domains. Other areas, such as industrial inspection, surveillance, and sports analytics, may exhibit distinct challenges that remain to be validated. Moreover, scenarios involving extreme low-light or night-time traffic conditions require further investigation, as do cases involving small or distant objects where more advanced multi-scale feature representations may be necessary. Finally, the present analysis has been limited to offline experiments on workstation-class GPUs. For safety-critical applications such as autonomous driving, systematic validation on embedded hardware platforms, together with the incorporation of complementary sensing technologies such as frame-based cameras, and compliance with real-time safety constraints, will be essential.

Future work will proceed along two complementary directions. On the algorithmic side, further developments such as lightweight attention mechanisms, enhanced multi-scale aggregation, and alternative temporal modules could strengthen the capacity of the model to capture fine-grained spatiotemporal patterns. On the systems side, deployment on embedded platforms including NVIDIA Jetson Xavier/Orin and FPGA-based accelerators will enable detailed profiling of throughput, latency, and energy consumption under realistic workloads. Such evaluations are critical for translating PMRVT from controlled experimental settings to practical deployment in domains such as autonomous driving, robotics, industrial inspection, and augmented reality.

## Figures and Tables

**Table 1:** Asymptotic complexity of key modules in RVT and PMRVT. Notation: *N* denotes the number of tokens, *d* the feature dimension, *P* the block size, and *G* the grid stride.

Module	RVT	PMRVT
Shallow encoder	$O(N^{2}d)$	$O(Nd^{2})$
Spatial attention	$O(N^{2}d)$	$O\left(NP^{2}+\frac{N}{P^{2}}d^{2}\right)+O\left(\frac{N}{G^{2}}d^{2}\right)$

**Table 2:** Comparison of different parameters and structural changes.

Stage	Size	Kernel	Stride	Channels		
				**PMRVT-T**	**PMRVT-S**	**PMRVT-B**
S1	1/4	7	4	32	48	64
S2	1/8	3	2	64	96	128
S3	1/16	3	2	128	192	256
S4	1/32	3	2	256	384	512

**Table 3:** Comparison of accuracy, parameter quantity, computational complexity, and inference time. The best results in accuracy are highlighted in bold. Inference time refers to the time it takes for the model to process input data and generate output results. Computational complexity (FLOPs) estimates the model’s computational burden. Data for RVT and PMRVT were obtained through actual measurements, while the rest were sourced from various papers. Best results in bold.

Method	Backbone	Detection Head	Gen 1			1 Mpx			Param
			**mAP (%)**	**Time (ms)**	**FLOPs (GMac)**	**mAP (%)**	**Time (ms)**	**FLOPs (GMac)**	
AsyNet [27]	-	-	14.5	-	-	-	-	-	11.4 m
AEGNN [12]	Graph Neural Network	YOLOX	16.3	-	-	-	-	-	20.0 m
MatrixLSTM [28]	RNN+CNN	YOLOv3	31.0	-	-	-	-	-	61.5 m
RED [7]	CNN+RNN	SSD	40.0	-	16.7	43.0	-	39.3	24.1 m
RT-DETR-B [18]	RT-DETR+ConvLSTM	YOLOX	47.6	-	15.8	45.2	-	42.7	42.8 m
S5-ViT-B [8]	-	-	47.4	-	8.16	47.2	-	9.57	18.2 m
GET [29]	Transformer	YOLOX	47.9	-	16.8	48.4	-	18.2	21.9 m
AEC [30]	Deformable-DETR	-	44.5	-	20.9	45.9	-	58.2	46.5 m
SAM [30]	ResNet50	-	35.5	-	6.0	23.9	-	19.0	>20 * m
Inception+SSD [31]	CNN	SSD	30.1	19.4	-	34.0	45.2	-	>60 * m
RRC-Events [7]	CNN	YOLOv3	30.7	21.5	-	34.3	46.4	-	>100 * m
YOLOv3Events [32]	CNN	YOLOv3	31.2	22.3	11.1	34.6	49.4	34.8	>60 * m
ASTMNet [33]	CNN+RNN	SSD	46.7	35.6	29.3	48.3	72.3	75.7	>100 * m
RVT [11]	Transformer+RNN	YOLOX	47.2	8.4	5.1	47.4	22.6	12.3	18.5 m
PMRVT (1 × 1) (ours)	Transformer+RNN	YOLOX	47.7	6.4	4.4	47.6	17.9	14.4	14.3 m
PMRVT (3 × 3) (ours)	Transformer+RNN	YOLOX	**48.7**	7.7	9.7	**48.6**	19.9	24.9	36.6 m

Note: * >x m indicates that the parameter count was reported as greater than x million by the respective papers, but the exact values were not provided.

**Table 4:** Ablation study of backbone block variants with 1 × 1 LSTM.

Block1	Block2	Block3	Block4	mAP	mAP_50_	mAP_75_	Time (ms)	Params (M)	FLOPs (GMac)
RVT	RVT	RVT	RVT	47.2	74.3	49.0	8.39	18.52	5.07
RVT	RVT	ATT	ATT	47.6	73.9	**49.7**	7.44	14.59	4.74
MLP	MLP	RVT	RVT	47.1	74.0	49.1	6.84	18.19	4.74
MLP	MLP	ATT	ATT	**47.7**	**74.5**	49.6	**6.40**	**14.26**	**4.40**

**Table 5:** Comparison of different kernel sizes for ConvLSTM.

	mAP	mAP_50_	mAP_75_	Time (ms)	Params (M)	FLOPs (GMac)
RVT (1 × 1) [11]	47.2	74.3	49.0	8.4	18.5	5.1
PMRVT (1 × 1)	47.7	74.5	50.0	**6.38**	14.3	4.40
PMRVT (3 × 3)	**48.7**	**76.2**	**50.9**	7.72	36.6	9.77

**Table 6:** Ablation study of ConvLSTM placement across stages.

S1	S2	S3	S4	mAP	mAP_50_	mAP_75_
			✓	37.5	62.1	38.2
		✓	✓	41.5	67.4	42.7
	✓	✓	✓	47.0	74.0	49.0
✓	✓	✓	✓	**48.7**	**76.1**	**50.9**

Note: ✓ indicates the stage where ConvLSTM is inserted.

**Table 7:** Cross-seed stability and statistical significance analysis of mAP (%) on Gen1 and 1 Mpx datasets. Results are reported as mean ± SD over 5 runs, with 95% CI in brackets. Δ denotes improvement over RVT, and *p*-values are computed via paired bootstrap testing.

Dataset	Model	mAP (Mean ± SD)	95% CI	Δ vs. RVT	*p*-Value
Gen1	RVT	37.2 ± 0.4	[36.6, 37.9]	–	–
	PMRVT (1 × 1)	38.3 ± 0.3	[37.8, 38.8]	+1.1	0.021 **
	PMRVT (3 × 3)	38.7 ± 0.5	[38.1, 39.5]	+1.5	0.008 **
1 Mpx	RVT	41.8 ± 0.6	[41.1, 42.7]	–	–
	PMRVT (1 × 1)	42.6 ± 0.4	[42.0, 43.3]	+0.8	0.049 *
	PMRVT (3 × 3)	43.0 ± 0.5	[42.4, 43.8]	+1.2	0.017 **

Note: * *p* < 0.05, ** *p* < 0.01.

**Table 8:** Robustness evaluation on Gen1 dataset under noise and event loss. mAP (%) is reported.

Model	Clean	+Noise (10%)	+Event Loss (20%)
RVT	37.2	34.8	32.9
PMRVT-1 × 1	38.3	36.7	35.5
PMRVT-3 × 3	38.7	37.4	36.1

## Data Availability

The datasets used in this study, including Gen1 and 1 Mpx event-camera datasets, are publicly available online. Gen1 is accessible at https://paperswithcode.com/dataset/gen1-detection (accessed on 28 September 2025) and 1 Mpx at https://paperswithcode.com/dataset/prophesee-gen4-dataset (accessed on 28 September 2025).
